# Modification of Ti6Al4V Titanium Alloy Surface Layer in the Ozone Atmosphere

**DOI:** 10.3390/ma12132113

**Published:** 2019-06-30

**Authors:** Mariusz Kłonica, Józef Kuczmaszewski

**Affiliations:** Department of Production Engineering, Faculty of Mechanical Engineering, Lublin University of Technology, 20-618 Lublin, Poland

**Keywords:** Ti6Al4V titanium alloy, ozone treatment, surface layer, surface free energy, adhesive joint

## Abstract

The paper reports the results of a study on the Ti6Al4V titanium alloy involving the XPS (X-ray photoelectron spectroscopy) photoelectron spectroscopy method. The position of bands in the viewing spectrum serves as a basis for the qualitative identification of atoms forming the surface layer, while their intensity is used to calculate the aggregate concentration of these atoms in the analyzed layer. High-resolution spectra are used to determine the type of chemical bonds based on characteristic numerical values of the chemical shift. The paper also presents the 3D results of surface roughness measurements obtained from optical profiling, as well as the results of energy state measurements of the Ti6Al4V titanium alloy surface layer after ozone treatment. It was shown that the ozone treatment of the Ti6Al4V titanium alloy removes carbon and increases concentrations of Ti and V ions at higher oxidation states at the expense of metal atoms and lower valence ions. The modification of the surface layer in ozone atmosphere caused a 30% increase in the Ti element concentration in the surface layer compared to the samples prior to ozone treatment. The carbon removal rate from the Ti6Al4V titanium alloy samples amounted to 35%, and a 13% increase was noted in oxides. The tests proved that the value of the surface free energy of the Ti6Al4V titanium alloy increased as a result of ozone treatment. The highest increase in the surface free energy was observed for Variant 4 samples, and amounted to 17% compared to the untreated samples, while the lowest increase was equal to 14%. For the analyzed data, the maximum value of standard deviation was 0.99 [mJ/m^2^].

## 1. Introduction

Titanium alloys are difficult to use in adhesive joining or other technologies where adhesive phenomena play a key role. This is due to a very wide variation in the properties of titanium oxides, depending on the oxidation conditions [1]. In industrial applications, particularly in the aerospace industry, the well-established electrochemical treatment methods for titanium alloys have been employed for years. However, these methods are cost-ineffective, also considering bath deposition. Chemical or electrochemical treatment technologies develop a properly constituted oxide layer. Although the obtained oxides typically exhibit high energy characteristics, they tend to be loosely attached to the substrate surface, which proves problematic considering adhesion processes. The growing interest in ozone treatment as an alternative method for imparting favorable energy conditions to the surface layer is an effect of high reactivity of ozone. The promising potential of this method may eventually lead to abandoning the well-established surface treatments, which, however, bear heavy environmental cost.

Searching for effective alternative methods for modifying adhesive properties [2] of titanium alloys is crucial from both the scientific and the practical perspective.

The energy state [3,4,5,6] of structural materials, especially titanium alloys in industrial applications, is particularly important in technologies where adhesion is crucial to process efficiency. Such technologies include adhesive joints, construction encapsulation, coating, printing, and sintering [1,7]. Proper bonding of structural materials requires not only suitable preparation of the substrate surface, but also developing adequate adhesion properties of adhesives or sealants [8,9,10].

Studies indicate that ozone treatment exhibits a promising potential [1,9] due to the fact that it increases the surface free energy. It is difficult to account for the phenomena that cause these changes as they take place at the nanoscale. Such changes may be observed at the macroscale only to a small extent by means of surface microscopy [11].

Chemical methods [12,13] and electrochemical pretreatment of the surface layer of structural materials are effective when removal of strongly bound material is required. They generate a suitable surface texture and provide high physicochemical activity of the surface with respect to the adhesive used.

The authors of the paper [14] used ozone to obtain titanium oxide. The results showed that this method produces a surface layer of the required properties.

This study set out to provide a more thorough chemical and topographic analysis of the titanium alloy surface following ozone treatment. The results offer an interesting insight into the changes occurring in the fusion layer and partly also in the surface layer of the cohesion zone of the Ti6Al4V alloy following the surface treatment in question.

## 2. Materials and Methods

The study was performed on the Ti6Al4V (Grade 5) titanium alloy according to the standards (AMS4911, ASTM B265). This alloy is widely used in the aviation, space and marine industries. The 25 mm × 100 mm × 1.6 mm Ti6Al4V titanium alloy samples were adhesively joined with the two-component epoxy adhesive, Loctite Hysol 9466. The adhesive was prepared by means of a static flow stirrer and applied to the adherend surfaces according to the manufacturer’s recommendations. Uniform thickness of the adhesive was ensured by the application of appropriate pressure during cure.

**Figure 1:**
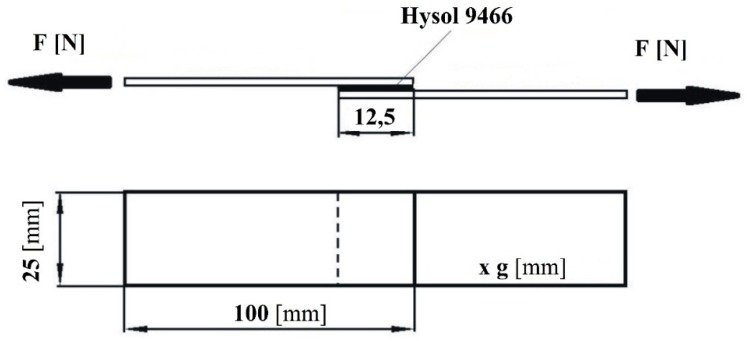
Single-lap adhesive joint: g-adherend thickness, F-force of the joint.

Figure 1 shows a schematic diagram of a single-lap adhesive joint.

The dimensional accuracy of the adherends was 0.1 mm, while that the length of the adhesive joint, 0.5 mm, with adhesive thickness gk = 0.1 ± 0.01 mm.

The adhesive was cured at an ambient temperature of 19 °C–22 °C, and relative humidity of 38–45%. The cure time was set to 120 h, and the unit pressure applied to the surface of the samples during bonding was equal to 0.2 MPa.

**Figure 2:**
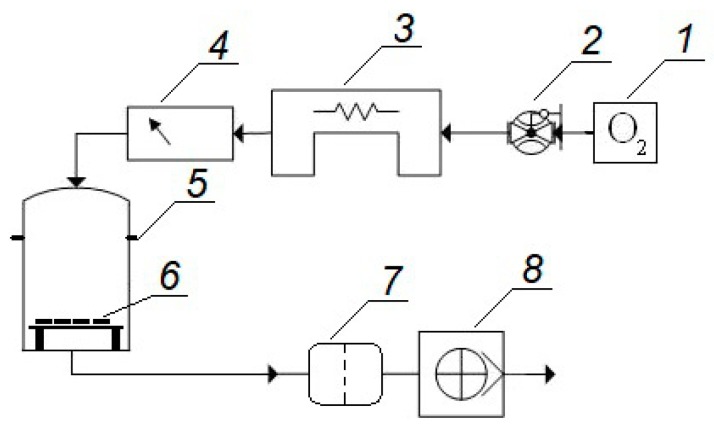
Layout diagram of test stand for ozone treatment of construction material samples: **1**—oxygen concentrator, **2**—adjustable flow meter, **3**—ozone generator, **4**—ozone concentration meter, **5**—reaction chamber, **6**—samples subjected to surface layer modification, **7**—ozone destroyer, **8**—suction pump.

The layout diagram illustrating the test stand for modification of the surface layer of construction materials in ozone atmosphere is shown in Figure 2.

The ozone flow in the ozone treatment process of samples was 0.9 dm^3^/min. The Ozone ANALYZER BMT 964 (BMT MESSTECHNIK GMBH, Stahnsdorf, Germany) was used for measuring ozone concentration.

The specimens were subjected to one of the four variants of ozone treatment, i.e.,

No. 1—samples after machining prior to ozone treatment,

No. 2—samples after machining and ozone treatment: 50g O_3_/m^3^ for 10 min,

No. 3—samples after machining and ozone treatment: 50g O_3_/m^3^ for 30 min,

No. 4—samples after machining and ozone treatment: 50g O_3_/m^3^ for 45 min.

The surface free energy (SFE) of the specimens was determined by means of the indirect method, based on the contact angle measurement. One of the most commonly used methods for determining the SFE of structural materials [3,4,5,6] is the Owens–Wendt method. This method assumes that the value of the SFE is the sum of its two constituents: The dispersive *γ_s_^d^* and the polar *γ_s_^p^* components of the SFE, and, in addition, that there is an additive relationship between these Equation (1):

(1) $$\gamma_{s}=\gamma_{s}^{d}+\gamma_{s}^{p},$$

where, *γ^s^*—the SFE of solids,

*γ_s_^d^*—the dispersive component of the SFE of tested substrates,

*γ_s_^p^*—the polar component of the SFE of tested substrates.

To calculate the dispersive and polar components of the SFE, of the tested substrate materials it is necessary to know the value of their contact angles. This is determined using the measuring liquids whose surface free energy, as well as polar and dispersive components, are known. In the presented tests, the measuring liquids were distilled water, as a polar liquid, and diiodomethane, as an apolar liquid. The dispersion component of the surface free energy is obtained from the given Equation (2):

(2) $${\left(\gamma_{s}^{d}\right)}^{0.5}=\frac{\gamma_{d}\left(\cos\Theta_{d}+1\right)-\sqrt{\frac{\gamma_{d}^{p}}{\gamma_{w}^{p}}}\gamma_{w}\left(\cos\Theta_{w}+1\right)}{2\left(\sqrt{\gamma_{d}^{d}}-\sqrt{\gamma_{d}^{p}\frac{\gamma_{w}^{d}}{\gamma_{w}^{p}}}\right)}$$

and the polar component of the surface free energy according is given by Equation (3):

(3) $${\left(\gamma_{s}^{p}\right)}^{0.5}=\frac{\gamma_{w}\left(\cos\Theta_{w}+1\right)-2\sqrt{\gamma_{s}^{d}\gamma_{w}^{d}}}{2\sqrt{\gamma_{w}^{p}}},$$

where, *γ_d_*—the SFE of diiodomethane,

*γ_d_^d^*—the dispersive component of the SFE of diiodomethane,

*γ_d_^p^*—the polar component of the SFE of diiodomethane,

*γ_w_*—the SFE of water,

*γ_w_^d^*—the dispersive component of the SFE of water,

*γ_w_^p^*—the polar component of the SFE of water,

*θ_w_*—the contact angle of water.

## 3. Results

**Figure 3:**
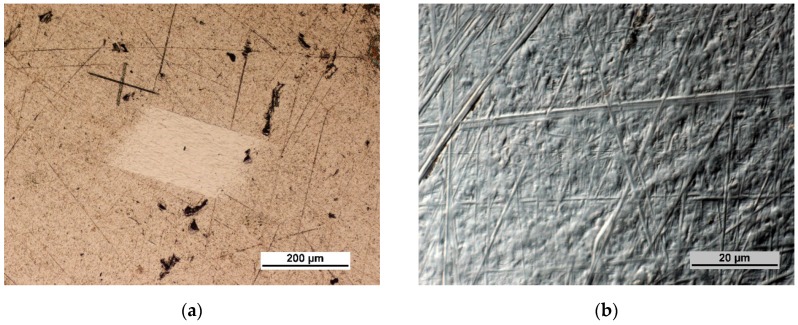
Ti6Al4V titanium alloy surface: (**a**) In polarized light at 50× magnification; (**b**) DIC image at 1000× magnification.

Photographs of the Ti6Al4V titanium alloy surface are shown in Figure 3a,b.

Figure 3a shows a fragment of the titanium alloy surface with a visible metallographic section produced by the ion abrasion technique obtained by means of a Quanta 3D FEG microscope (FEI, Hillsboro, OR, USA). A cross-shaped marker is also visible. The marker was used to identify the place of measurements before and after ozone treatment. Precise measurements enabled recording the changes occurring at the same place of the surface layer following ozone treatment.

### 3.1. Surface Roughness

The surface roughness was measured by means of optical profilometry using a Contour GT-K1 optical profilometer (Veeco, Bruker, the Netherlands). The purpose of the study was to determine the effect of surface layer modification in the ozone atmosphere on the surface layer of structural materials. The following parameters were measured in the experiments: Sa—arithmetic mean of the 3D profile ordinates, Sq—root mean square of 3D profile ordinates, Sp—value of the highest elevation of 3D profile, Sv—value of the lowest 3D profile depth and Sz—maximum height of the 3D profile.

The paper [10] pointed to the significance of surface topography on the strength of lap adhesive joints and investigated the surface roughness parameters that affect the strength of adhesive joints.

**Figure 4:**
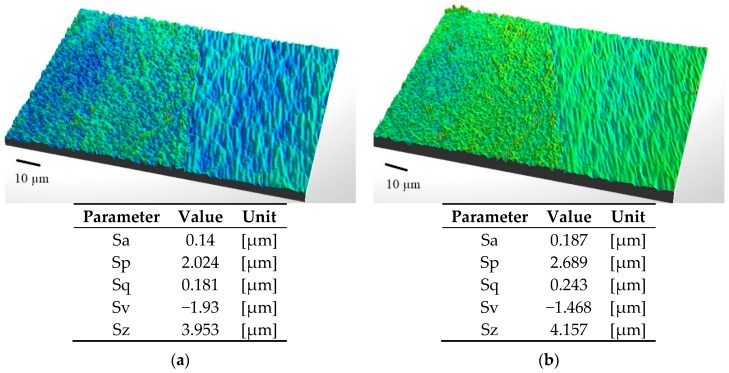
Images of Ti–6Al–4V titanium alloy samples surface with clear limit after ionic polishing: (**a**) Before ozone treatment; (**b**) after ozone treatment (samples after ozone treatment: 50 g O_3_/m^3^ for 45 min).

Figure 4 shows the topography of Ti6Al4V titanium alloy samples before and after ozone treatment. The size of the scanned surface is 156 μm × 117 μm.

The results indicate an over 25% increase in the surface roughness of the entire joint area after ozone treatment compared to the untreated surface. The value of the roughness parameter Sq before pretreatment, 0.181 μm, increases to 0.243 μm after the ozone treatment. Similar changes are observed in other parameters after treatment.

The experimental results demonstrate that ozone “interacts” with the smooth surface of this alloy.

### 3.2. Examination of the Surface Layer with Electron Scanning Microscopy

**Figure 5:**
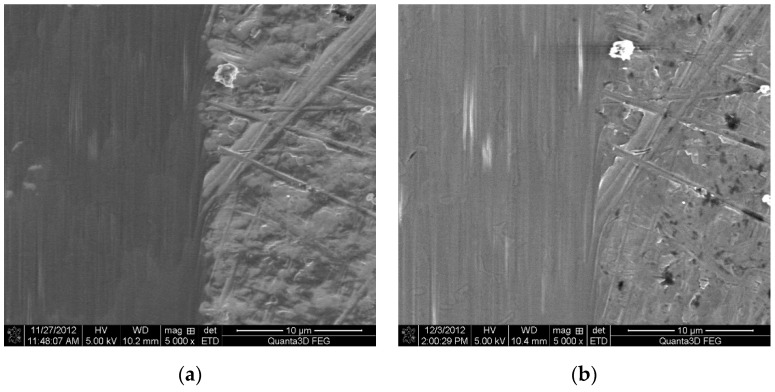
Topography of Ti6Al4V titanium alloy surface after ionic polishing with FIB (focus ion beam) technique: (**a**) Before ozone treatment; (**b**) after ozone treatment (samples after ozone treatment: 50 g O_3_/m^3^ for 45 min).

Figure 5 shows photographs of untreated Ti6Al4V titanium alloy surface and after modification of the surface layer in ozone atmosphere. The figures show examples of areas after ionic polishing, prior to and following ozone treatment.

The surface layer of the samples was examined using a high-contrast scanning electron microscope (Quanta 3D FEG; FEI, Hillsboro, OR, USA), which enables registration of secondary electrons (SE). The maximum magnification was 5000×.

The obtained increase in the values of surface roughness parameters following ozone treatment is notable on nano-scale. The effect is complex, certain micro-irregularities of the surface may become leveled out, whereas new micro-cavities may be formed on the treated surface, which effects in the rise in surface roughness parameters.

The results of the surface examination are shown in Table 1. Table 1 shows the results for Ti6Al4V titanium alloy before and after ozone treatment. The scanning area was set to 1 mm^2^.

The ozone treatment of Ti6Al4V titanium alloy effectively removes carbon from the surface of contaminated samples. The removal of atmospheric carbon leads to a percentage increase of other Ti6Al4V titanium alloy components.

### 3.3. Examination of the Surface Layer with XPS Photoelectron Spectroscopy

The results of XPS and FTIR (Fourier Transform Infrared Spectroscopy) analyses [15] reveal the existence of an oxidized polymer layer in ozone-treated samples and the effect of ozone on polar bonds such as C‒O or C=O.

The XPS results reported in the paper [16] show irreversible changes in the surface layer of titanium alloy under the impact of ozone. The results analyzed in [16], however, concern the human body environment, which exhibits totally different conditions. The decrease in the surface carbon content is a result of the cleaning effect of ozone, while the increase in oxygen levels in the surface layer indicates the formation of oxides. Simultaneously, the analyses show that the content of pure titanium decreases.

**Figure 6:**
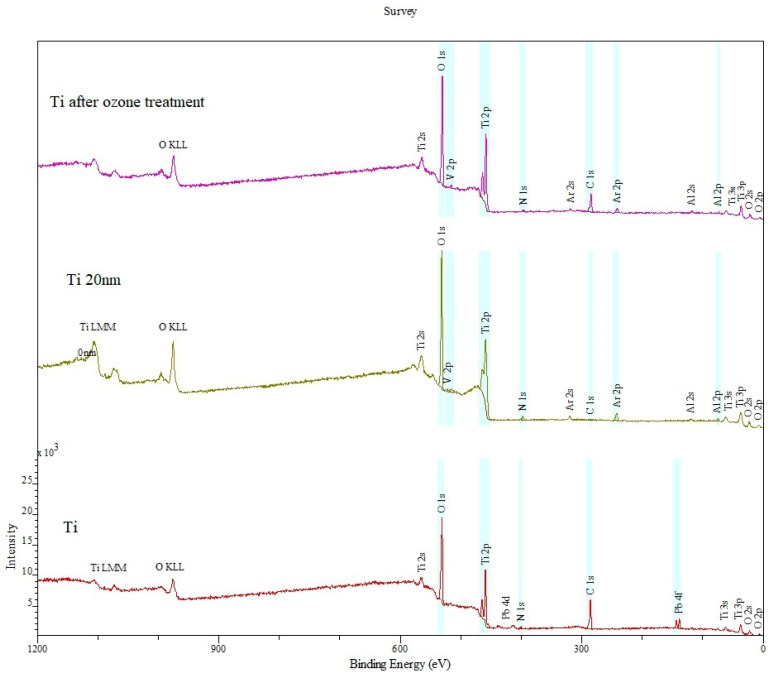
Comparative kit of XPS broadband spectra of Ti6Al4V titanium alloy samples in the following order: A spectrum of titanium alloy before ozone treatment (Ti), after plasma cleaning (Ti 20 nm) and after ozone treatment (Ti after ozone treatment).

**Figure 7:**
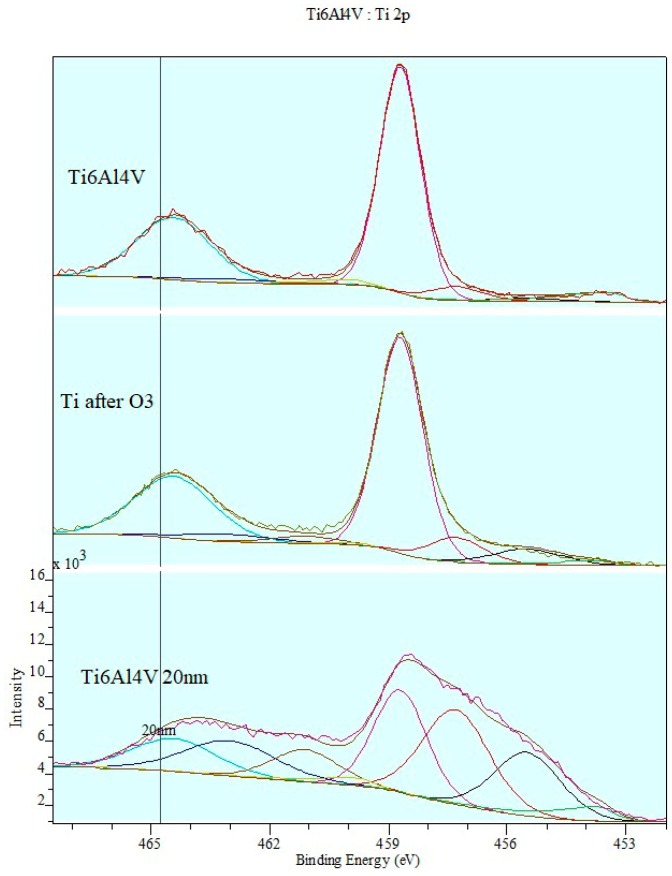
Changes in doublets at valence band in Ti2p spectrum.

**Figure 8:**
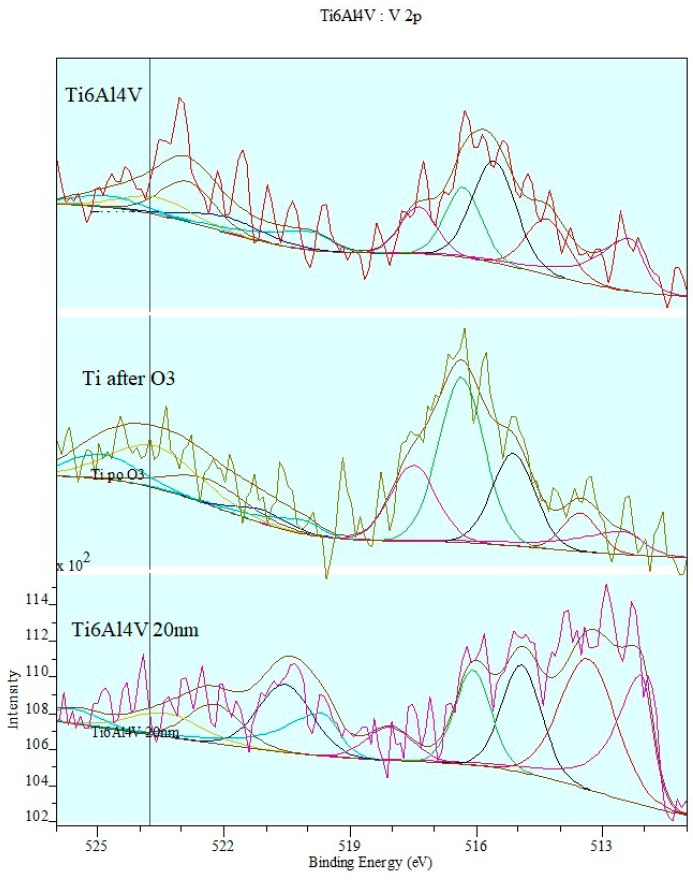
Effect of ozone treatment on XPS spectrum on the basis of V2p transitions.

**Figure 9:**
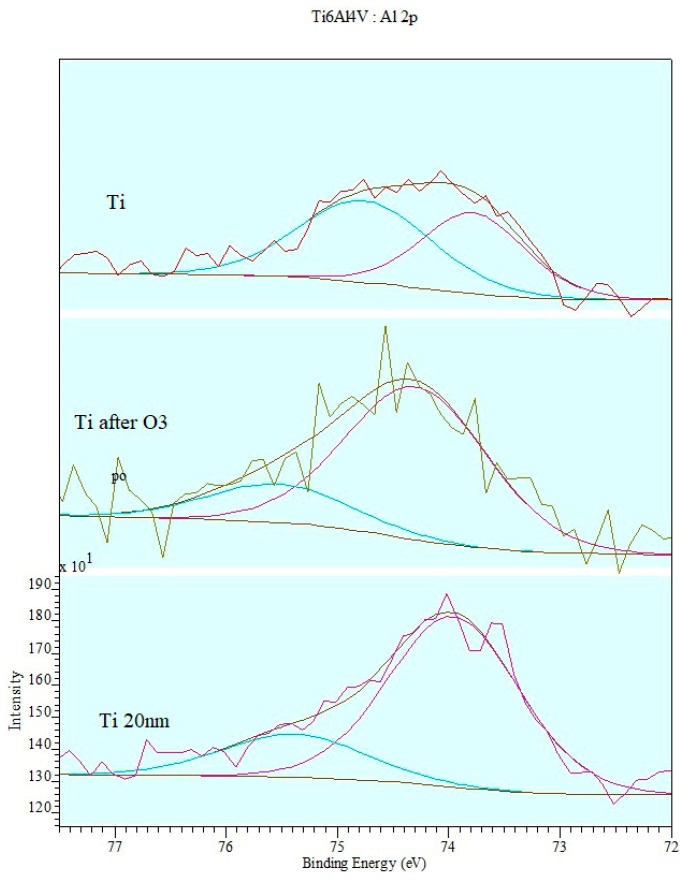
Deconvolution of XPS spectrum of Al2p band: Titanium alloy before ozone treatment (Ti), titanium alloy after ionic cleaning (Ti 20 nm) and titanium alloy after ozone treatment (Ti after ozone treatment).

**Figure 10:**
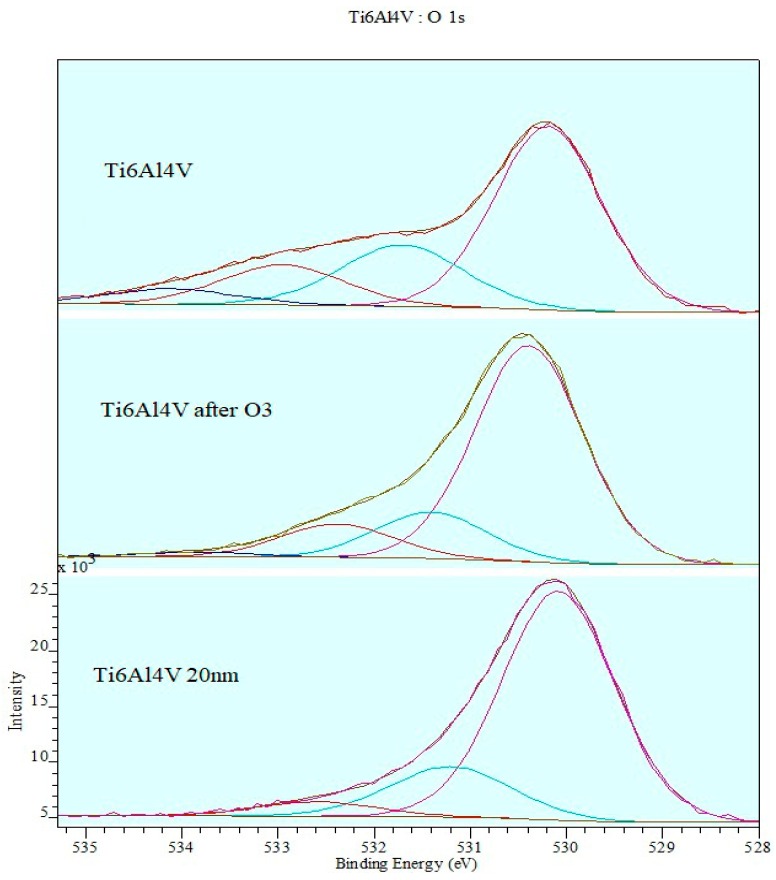
Deconvolution of XPS spectrum of O1s band.

**Figure 11:**
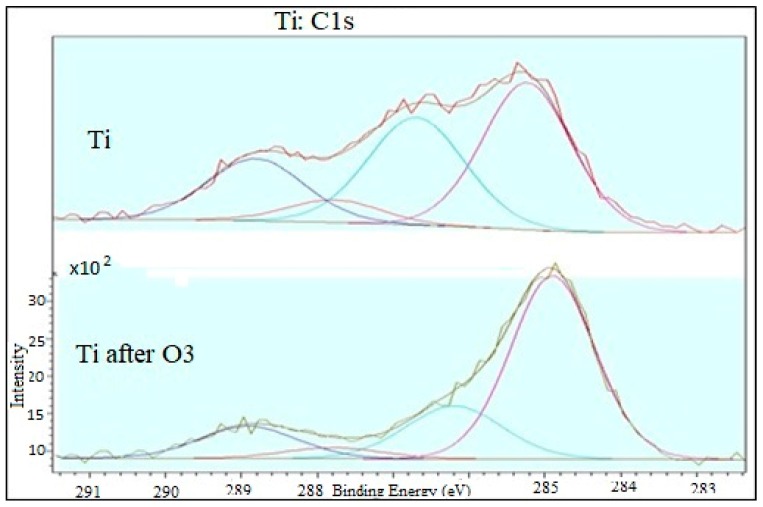
Matching XPS spectrum to C1s band.

**Figure 12:**
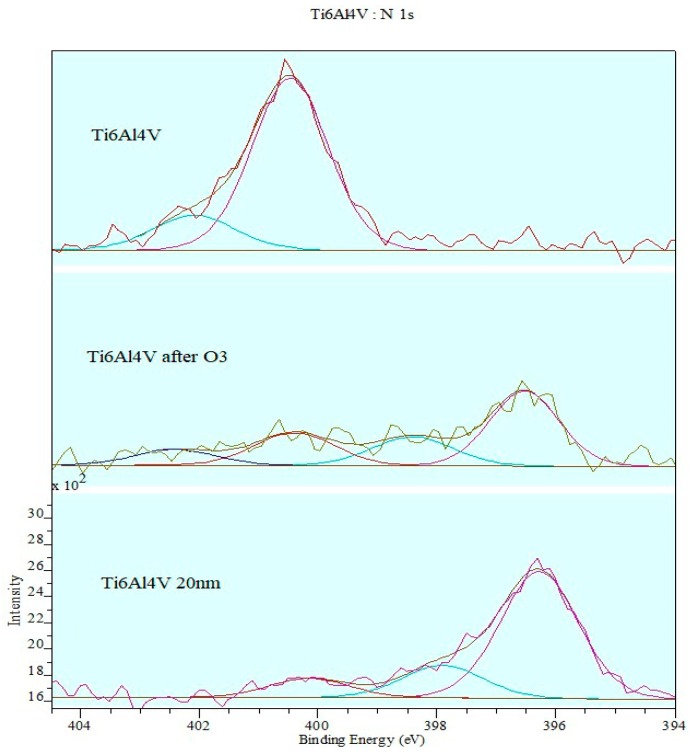
XPS spectrum of photoelectrons in titanium alloy sample.

Figure 6, Figure 7, Figure 8, Figure 9, Figure 10, Figure 11 and Figure 12 show the results of Ti6Al4V titanium alloy surface composition examination. The surface layer of this alloy is highly sensitive to contamination. Despite washing and degreasing before ozone treatment, the alloy was contaminated with a layer of surface carbon and lead. The concentration of titanium alloys constitutes approx. 31% of its nominal weight value, 85% of which is in the oxidized form.

A comparative kit of XPS wideband spectra of the Ti6Al4V titanium alloy sample is shown in Figure 6.

What can be seen in the high-energy part of the XPS spectrum are wide KLL Auger bands of oxygen (EB ~980 eV) and LMM Ti (EB ~1120 eV). The titanium alloy surface is deficient in vanadium atoms, which remains even after ionic cleaning of the adherend. The cleaning of the adherend surface and removing 20 nm of the layer led to a considerable change in surface composition of the alloy. Lead was entirely removed from the surface layer of the specimen, and so was almost entire surface carbon. The removal of the carbon layer exposes the grains of aluminum and titanium. As a result of ozone treatment, C content on the surface of Ti6Al4V dropped by approx. 35%.

Table 2 shows the chemical composition of Ti6Al4V titanium alloy substrate prior to and following ozone treatment.

Pb content on the surface of specimens prior to treatment is an effect of ambient environment contamination. Ionic and ozone treatment remove PB entirely.

Figure 7 shows changes in the doublets at the valence band in the Ti2p spectrum following Ti6Al4V titanium alloy surface modification.

The ozone treatment of the Ti6Al4V titanium alloy slightly changes the shape of the composite band and the intensity of its components. What can be noticed comparing the spectral band profiles of Ti2p alloy after ionic etching and ozone treatment is deep ozonation of the adherend surface layer. What is also notable is the drop in the intensity of metallic phase peaks in the spectrum as well as Ti (II) and Ti (III) ions in a low-energy XPS part of the spectrum. The peak located at EB = 459 eV bound to TiO2 titanium oxide at the highest oxidation state is the dominant band. Ti (IV) form amounts to 79% of total concentration after ozone treatment, which is more than a two-fold increase, compared to the 28% concentration after ionic cleaning.

Figure 8 shows the effect of ozone treatment on XPS spectrum based on V2p transitions.

The course and nature of the observed changes clearly indicate the oxidation process of the surface layer of the titanium alloy sample during ozone treatment. Unlike titanium ions, the ozone treatment process essentially changes the shape of the composite band and the distribution of matching components. The concentration of forms with a higher oxidation degree: V(IV) and V(V) significantly increases at the expense of metal and V(II) ions.

The deconvolution of the XPS spectrum for the Al2p band is shown in Figure 9.

The deconvolution procedure of the band reveals two phases of aluminum oxide on the alloy surface. After ozone treatment, aluminum oxide Al_2_O_3_ constitutes the main phase with the 80% content of Al.

The deconvolution procedure of the XPS spectrum for the O1s band (Figure 10) confirms the coexistence of various oxides and a high degree of oxidation of the surface layer of the alloy after ozone treatment. The intensity axis was normalized.

Figure 11 depicts the deconvolution of the XPS spectrum of the C1s band.

The deconvolution procedure of the band confirms the coexistence of oxidized and non-oxidized forms of surface carbon.

Figure 12 shows the XPS spectrum of photoelectrons in the Ti6Al4V titanium alloy sample.

After the deconvolution of the peaks on the spectrum, we may distinguish bands of N1s photoelectrons that come from three chemically different phases.

The results from the chemical analysis show that the surface layer of the alloy is enriched with aluminum atoms, as their concentration, over 6 wt %., exceeds the nominal value, and which occur in the entirely oxidized form as Al_2_O_3_ oxide, Al(OH)_3_ hydroxides and AlO(OH) boehmite. The spectral analysis results, furthermore, reveal that argon permanently penetrates the crystal lattice and is not removed in high vacuum of the UHV system. The adherend also contains nitrogen which can form various chemical bonds.

The results clearly show that the surface layer of the titanium alloy before ozone treatment is practically entirely covered with a layer of metal oxides, showing different degrees of oxidation and contaminated with the carbon layer. The tests show that the ozone treatment of titanium alloy results in the removal of carbon and the increase in the concentration of Ti and V ions in an advanced stage of oxidation at the expense of metal atoms and ions with lower valence. The modification of the surface layer in the ozone atmosphere resulted in a 30% increase of Ti element concentration in the sample surface layer compared to the samples prior to the ozone treatment. The removal of carbon from Ti6Al4V titanium alloy samples during the ozone treatment process is estimated at 35%. A 13% increase in oxides after ozone treatment was also observed.

### 3.4. Surface Free Energy

**Figure 13:**
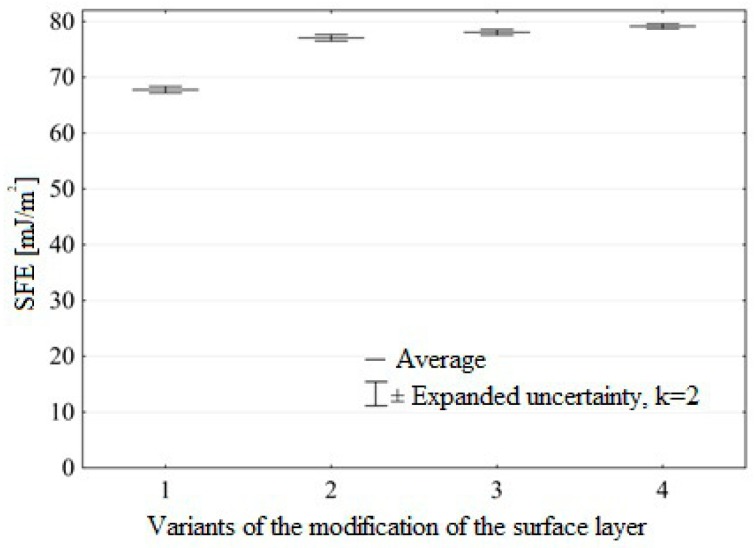
Surface free energy of Ti6Al4V titanium alloy for different ozone treatment conditions (modification variants of surface layer are described in “Materials and Methods”).

Figure 13 shows the surface free energy of Ti6Al4V titanium alloy after machining using a density tool with a grit size of P320.

The results demonstrate that the surface free energy of Ti6Al4V titanium alloy increased as a result of the ozone treatment. The highest increase in the surface free energy was observed for the samples prepared in accordance with the fourth variant of surface pretreatment, amounting to 17% compared to the samples before ozone treatment. The lowest observed increase was 14%. For the analyzed data, the maximum value of standard deviation was 0.99 [mJ/m^2^].

**Figure 14:**
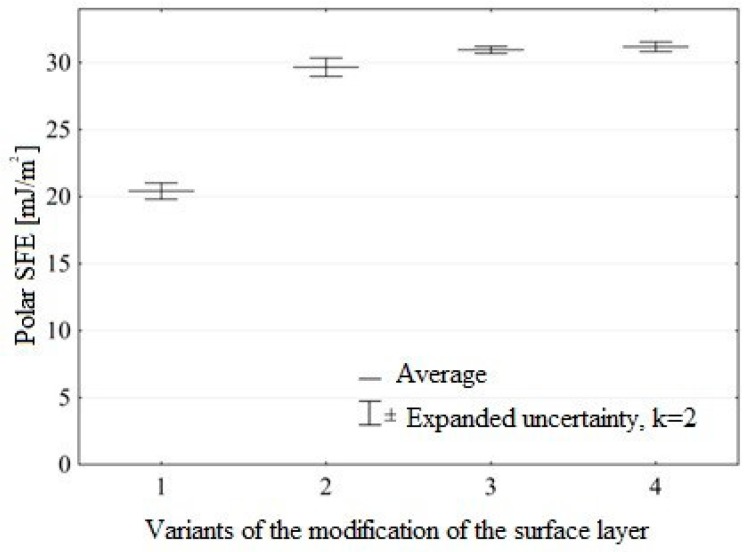
Polar component SFE of Ti6Al4V titanium alloy for different ozone treatment conditions (modification variants of surface layer are described in “Materials and Methods”).

Compared to the samples prior to ozone treatment, the highest increase in the polar component of the SFE of the Ti6Al4V titanium alloy after pretreatment (Figure 14) was 51%, while the lowest increase was by 44%.

The results clearly demonstrate that modification of the surface layer of the tested construction materials in ozone atmosphere has a considerable effect on increasing the surface free energy and its polar component.

### 3.5. Strength of the Adhesive Joint

**Figure 15:**
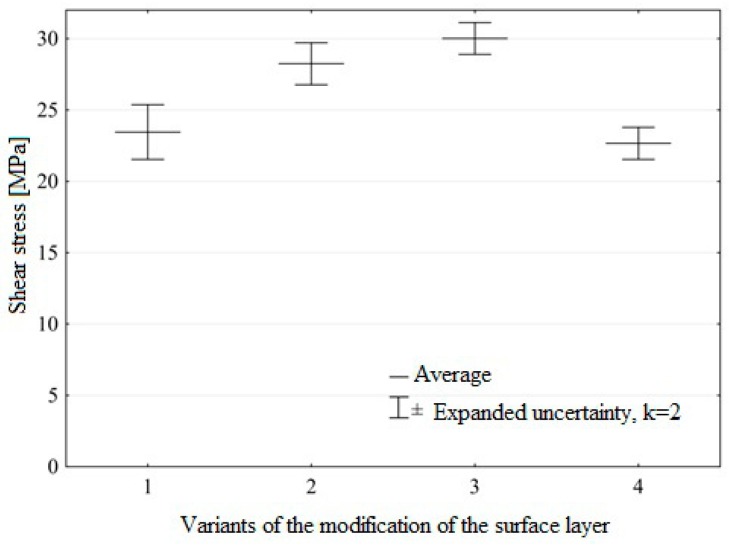
Shear stress at obtained in experimental studies for Ti6Al4V titanium alloy samples made with Hysol 9466 adhesive, for various ozone treatment conditions (modification variants of surface layer are described in “Materials and Methods”).

Figure 15 shows mean values of shear stresses in a single-lap adhesive joint obtained in experimental studies for the Ti6Al4V titanium alloy after machining and ozone treatment.

The ozone treatment process of Ti6Al4V titanium alloy samples increases to some extent the strength of the single-lap adhesive joint made with the Hysol 9466 adhesive. The highest increase in strength of the adhesive joint, amounting to 28%, was observed for ozone samples with a concentration of 50 g O_3_/m^3^ in 30 minutes. In this case, the extreme has been reached. The decrease in strength observed for Variant 4 confirms the significant effect of the ozone treatment process on the final effects.

## 4. Conclusions

The findings of this study have led to the following conclusions:

(1) Ozone treatment of the titanium alloy results in the removal of carbon and an increase in the concentration of Ti and V ions at higher oxidation states at the expense of metal atoms and ions with lower valence.
(2) The modification of the surface layer in ozone atmosphere caused a 25% increase in the Ti element of the surface layer of the samples compared to the samples before ozone treatment.
(3) Removal of carbon from Ti6Al4V titanium alloy samples in the ozone treatment process amounted to 35%, and the content of oxides increased by 13%.
(4) The increase in the surface free energy was observed in the Ti6Al4V titanium alloy as a result of the ozone treatment, the highest increase in the surface free energy was observed for the samples prepared according to Variant 4 and increased by 17%, compared to the untreated samples, while the smallest increase was 14%.
(5) There is no correlation between the highest increase in the surface free energy and an increase in the strength of adhesive joints. The knowledge on the surface free energy value is, therefore, a necessary yet insufficient condition for designing predictive models of adhesive joints strength of metal substrates.
(6) The highest increase in the strength of adhesive joint was noted for the samples subjected to ozone treatment with a concentration of 50 g O_3_/m^3^ over 30 minutes. The untreated samples exhibited an increase of 28%.

The study confirms that ozone treatment, if performed under appropriate conditions, can be used in bonding technologies to shape surface micro-topography and free energy, thus offering an alternative option to electrochemical methods.

## Figures and Tables

**Table 1:** Results of EDX (energy dispersive X-ray spectroscopy) analysis of Ti6Al4V titanium alloy sample before and after ozone treatment.

Element	Sample before Ozone Treatment		Sample after Ozone Treatment: 50 g O_3_/m^3^ for 45 min	
	wt % (weight)	at % (atom)	wt % (weight)	at % (atom)
Al	6.13	10.20	6.08	10.13
Ti	89.14	83.53	89.11	83.65
V	3.61	3.18	3.73	3.29
Fe	0.22	0.17	0.25	0.21
Other	0.9	2.92	0.84	2.72

**Table 2:** The chemical composition of Ti6Al4V titanium alloy prior to and after ozone treatment.

Element	Ti6Al4V Titanium Alloy Prior to Ozone Treatment				Ti6Al4V Titanium Alloy after Ozone Treatment			
	%	at %	Fractions at %		%	at %	Fractions at %	
Ti	28.23	12.15	Ti (0)	6.74	37.72	16.86	Ti (0)	2.21
			Ti (II)	1.75			Ti (II)	8.01
			Ti (III)	6.59			Ti (III)	10.72
			Ti (IV)	84.92			Ti (IV)	79.06
V	1.86	0.75	V (0)	21.76	0.96	0.40	V (0)	9.14
			V (II)	15.55			V (II)	8.64
			V (III)	32.00			V (III)	19.77
			V (IV)	18.18			V (IV)	33.26
			V (V)	12.51			V (V)	29.19
Al	4.89	3.73	Al (III)	100	4.44	3.52	Al (III)	100
C	23.78	40.78	-		15.56	27.72	-	
O	31.42	40.46	-		35.55	47.56	-	
Pb	8.98	0.89	-		-	-	-	
